# Soft metamaterials with dynamic viscoelastic functionality tuned by pre-deformation

**DOI:** 10.1098/rsta.2018.0072

**Published:** 2019-03-18

**Authors:** William J. Parnell, Riccardo De Pascalis

**Affiliations:** 1 School of Mathematics, University of Manchester, Oxford Road, Manchester M13 9PL, UK; 2 Dipartimento di Matematica e Fisica ‘E. De Giorgi’, Università del Salento, 73100 Lecce, Italy

**Keywords:** viscoelasticity, incremental deformations, effective moduli

## Abstract

The small amplitude dynamic response of materials can be tuned by employing inhomogeneous materials capable of large deformation. However, soft materials generally exhibit viscoelastic behaviour, i.e. loss and frequency-dependent effective properties. This is the case for inhomogeneous materials even in the homogenization limit when propagating wavelengths are much longer than phase lengthscales, since soft materials can possess long relaxation times. These media, possessing rich frequency-dependent behaviour over a wide range of low frequencies, can be termed *metamaterials* in modern terminology. The sub-class that are periodic are frequently termed *soft phononic crystals* although their strong dynamic behaviour usually depends on wavelengths being of the same order as the microstructure. In this paper we describe how the effective loss and storage moduli associated with longitudinal waves in thin inhomogeneous rods are tuned by pre-stress. Phases are assumed to be quasi-linearly viscoelastic, thus exhibiting time-deformation separability in their constitutive response. We illustrate however that the effective incremental response of the inhomogeneous medium does *not* exhibit time-deformation separability. For a range of nonlinear materials it is shown that there is strong coupling between the frequency of the small amplitude longitudinal wave and initial large deformation.

This article is part of the theme issue ‘Rivlin's legacy in continuum mechanics and applied mathematics’.

## 1. Introduction

Visco-elastomeric materials are employed in numerous applications, e.g. noise and vibration isolators in machinery and the automotive and aerospace industry, bridge bearings and seismic shock absorbers in civil engineering applications as well as soft robotics, artificial muscles and more general soft tissue modelling [1–3]. In these contexts, the materials are frequently subjected to pressures that give rise to large deformations of the medium. In the soft tissue context, a pre-stress may exist due to some existing residual stress or it could be induced by physiological or mechanical mechanisms, e.g. for medical purposes in order to increase tissue contrast prior to scans [4,5]. It is frequently useful to study the subsequent *incremental* (linear) response of elastomeric and visco-elastomeric materials in order to assess the stability, incremental constitutive behaviour or simply the fitness-for-purpose of the materials in question. The associated theory is now commonly referred to as *small-on-large theory* [6–8]. Synthetic materials for the applications referred to above are becoming ever more complex, and it is common now for these media to be composite in their nature, comprising at least two phases combined in some pre-determined manner. The composite usually aims to optimize, or at least enhance, some aspect of material behaviour [9,10]. As a result, it is important to understand the effective incremental properties of the medium in such pre-stressed states. This also helps to build a picture of the effective nonlinear behaviour of the medium in question.

Extensive work has been done on predicting the effective linear response of such inhomogeneous materials when they are in an *unstressed* state. In order to be able to define an *effective* material, the macroscopic lengthscale (i.e. the lengthscale over which macroscopic deformations vary) must be much longer than the microstructural lengthscale. In this regime, effective elastic moduli, viscosities and other mechanical properties can be derived by using homogenization methods and micromechanical techniques [11–13]. In particular when the microstructure is distributed *periodically*, closed-form solutions can often be found [14–17]. The field of metamaterials is related to, but rather distinct from, this homogenization scenario in the sense that a metamaterial can give rise to a frequency-dependent response even in this low-frequency (homogenization) limit, usually being associated with an induced resonance of the microstructure [18,19]. Alternatively, a metamaterial can be more straightforward in the sense that it could slow down or redirect sound [20–23], and these properties are normally induced by complex microstructure in the homogenization limit.

Over the last decade, pre-stressed nonlinear materials have been employed to good effect in a number of scenarios, e.g. hyperelastic cloaking, elastodynamic redirection [24–27] and band-gap tuning [28–37], thereby having a significant influence on the field of tuneable and configurable metamaterials. What has not, however, been studied in these scenarios is the impact of loss, or viscous mechanisms, even though these materials are inherently lossy, although a mechanism to balance loss mechanisms by employing time dependent material properties was recently proposed in the layered medium context [38]. Furthermore, as is well known, viscoelastic materials often present a range of frequency-dependent behaviour even in the low-frequency regime and this therefore represents an alternative metamaterial property that has not yet been investigated or exploited.

A variety of models have been suggested for the behaviour of nonlinear viscoelastic materials. Modelling such media requires an explicit time-dependent model to be formulated [39]. In recent times, Fung's theory of quasi-linear viscoelasticity (QLV) has been revisited given its (relative) ease of implementation for large deformation problems [40–43], and also given its potential to be employed for problems associated with compressible media [44] and for anisotropic materials with distinct relaxation responses in principal directions [45]. The isotropic QLV theory is essentially equivalent to the theory of Simo, based on internal variables and evolution equations in the incompressible limit [46].

As referred to above, small-on-large (SOL) theory is a classical theory developed predominantly over the second half of the twentieth century in the context of nonlinear elasticity, allowing the study of stability under large deformation and also wave propagation in pre-stressed states [7]. Similarly, theories have been presented for viscoelasticity but the more complex constitutive response makes such a study rather complex and the conclusions depend strongly on the viscoelastic constitutive response chosen. Rivlin was a pioneer in this field, developing a general theory of SOL viscoelasticity with Pipkin for materials with fading memory [47], which allows one to write down a general expression for the incremental stress in a medium subject to large deformation in either a non-steady or steady configuration. Rivlin's subsequent work with Hayes employed the Rivlin–Ericksen constitutive form to lay down the foundations for the study of the propagation of small-amplitude viscoelastic waves in a homogeneously deformed medium [48–50].

Zapas and Wineman considered small oscillatory torsional deformations superposed on a uniform extension with the BKZ constitutive model [51], motivated by a classical result of Rivlin [52], who showed that in the elastic case the superposed torsional stiffness is independent of the strain energy density function and can be expressed entirely in a known relationship between the axial force and the axial stretch ratio. Zapas and Wineman showed that viscoelastic oscillations associated with the BKZ model breaks this independence.

For reasons of ease of implementation, as described above, there is great interest in using the Simo/QLV models of nonlinear viscoelasticity. Recall that these theories exhibit strain independent relaxation and therefore the incremental equations give rise to incremental stress–strain relations that are time-deformation separable [53,54]. In a related theory, Morman and Nagtegaal proposed a simplified theory of SOL viscoelasticity which was time-deformation separable [55]. It is known however that although this assumption is often reasonable for *homogeneous* materials, heterogeneous materials respond rather differently and the so-called *effective relaxation function* of the composite can be strongly dependent on stretch. Posing a functional form for this relaxation function however is non-trivial. In order to deal with this issue, Kim *et al.* introduced a so-called *static deformation correction factor* into the effective relaxation function [53,54], although this manner of introduction and the choice of its functional dependence are rather arbitrary. Here, assuming time-deformation separability for homogeneous phases of a composite rod with simple geometry (piecewise constant properties), we illustrate how the effective incremental response depends on both pre-stretch and time (frequency). Using homogenization theory in this pre-stressed state one can derive the manner in which the effective incremental linear properties depend on the pre-deformation.

It should be clear from the above discussion that Ronald S. Rivlin provided the foundational theoretical work in the area of viscoelastic waves in pre-stressed materials. We hope that the present work illustrates just one aspect of the legacy of Rivlin's work, i.e. the importance of theoretical work associated with wave propagation in pre-stressed nonlinear materials. It shows that his work has had a lasting influence today and impacts on fields such as nonlinear viscoelastic materials and metamaterials that have the potential for far-reaching impact in a number of fields of modern science and technology.

The paper proceeds as follows: in §2, we describe the formulation of linear viscoelasticity in the time and subsequently in the frequency domain. Following this in §3, we illustrate how one can employ homogenization theory in order to derive the effective viscoelastic Young's modulus and in particular that this (complex) modulus can have strong frequency dependence even in the homogenization regime due to its relaxation spectrum. This is particularly the case for soft materials, which tend to have long relaxation times. In §4, we describe the finite deformation of nonlinear viscoelastic homogeneous materials and the relation of the quasi-static long time limit of a uniaxial deformation to the associated hyperelastic deformation problem. In §5, we then formally derive the incremental equation governing longitudinal waves in a homogeneous, thin incompressible quasi-linearly viscoelastic rod that has been subjected to uniaxial tension, inducing large time-dependent deformations. We show that in this homogeneous case the incremental equation has coefficients that are frequency-deformation separable. In §6, we then study inhomogeneous rods to determine the effective *viscoelastic* Young's modulus in the pre-stressed state, assuming that each homogeneous phase has time-deformation separability. Importantly, it is shown that the effective (homogenized) incremental response does *not* behave in this separable manner with strong coupling between frequency and stretch.

## 2. Linear viscoelasticity

Consider the most general linear viscoelastic constitutive relation, in the form [39,56]

2.1 $$\hat{\sigma }(t)=\int_{-\infty }^{t}\hat{G}(t-\tau ):d\hat{e}(\tau )=\int_{-\infty }^{t}\hat{G}(t-\tau ):\frac{\partial \hat{e}(\tau )}{\partial \tau }\;d\tau ,$$

where $\hat{\sigma }$ and $\hat{e}$ are the (second order) linearized stress and strain tensors, respectively, while $\hat{G}(t)$ is the (fourth-order) stress relaxation tensor, noting that ':' indicates double contraction. One can also write down this constitutive relation in its inverse form, i.e.

2.2 $$\hat{e}(t)=\int_{-\infty }^{t}\hat{J}(t-\tau ):d\hat{\sigma }(\tau )=\int_{-\infty }^{t}\hat{J}(t-\tau ):\frac{\partial \hat{\sigma }(\tau )}{\partial \tau }\;d\tau ,$$

where $\hat{J}(t)$ is the *creep* compliance tensor. In this paper however attention will be focused on (2.1). The constitutive equations (2.1) and (2.2) are written in the form of a time-convolution, which takes into account any jump discontinuities in the arguments of strain or stress. When the motion starts at the instant *t* = 0, (2.1) can be written as [56]

2.3 $$\hat{\sigma }(t)=\hat{G}(t):\hat{e}(0)+\int_{0}^{t}\hat{G}(t-\tau )\frac{\partial \hat{e}(\tau )}{\partial \tau }\;d\tau .$$

Integrating by parts, this becomes

2.4 $$\hat{\sigma }(t)=\hat{G}(0):\hat{e}(t)+\int_{0}^{t}\frac{d\hat{G}(t-\tau )}{d(t-\tau )}:\hat{e}(\tau )\;d\tau .$$

Let us consider now the constitutive law governing *isotropic* linear viscoelastic materials. The relaxation tensor then takes the form

2.5 $$\hat{G}(t)=3\hat{\kappa }(t)I^{1}+2\hat{\mu }(t)(I^{2}-I^{1}),$$

where $\hat{\kappa }(t)$ and $\hat{\mu }(t)$ are two independent relaxation functions and the fourth-order basis tensors **I**^1^ and **I**^2^ have Cartesian components defined as

2.6 $$I_{ijkl}^{1}=\frac{1}{3}\delta_{ij}\delta_{kl}\;\mathrm{and}\;I_{ijkl}^{2}=\frac{1}{2}(\delta_{ik}\delta_{jl}+\delta_{jk}\delta_{il}).$$

Employ (2.5) in (2.3) and apply the Fourier transform (referring to appendix A), defined for some function $\hat{f}(t)$ by

2.7 $$f(\omega )=\int_{-\infty }^{\infty }\hat{f}(t)\;e^{i\omega t}\;dt$$

noting that the time-domain function has the hat and the transform domain function does not. We find that in the Fourier transform domain the isotropic form of (2.3) can then be written as

2.8 $$\sigma (\omega )=3K(\omega )e_{H}(\omega )+2M(\omega )e_{D}(\omega )$$

where ***e***_h_ = (1/3) tr(***e***)**I** and ***e***_d_ = ***e*** − ***e***_h_, where **I** is the second-order identity tensor with Cartesian components *δ*_*ij*_ and where we assume for the sake of convergence that Im(*ω*) > 0. Furthermore, K(ω) and M(ω) are the frequency domain moduli:

2.9 $$K(\omega )=-i\omega \kappa^{+}(\omega )\;\mathrm{and}\;M(\omega )=-i\omega \mu^{+}(\omega )$$

where a superscript + denotes the half-range Fourier transform

2.10 $$f^{+}(\omega )=\int_{0}^{\infty }\hat{f}(t)\;e^{i\omega t}\;dt.$$

Experimentally, it is often found that to a good approximation the Poisson ratio *ν* of a wide class of viscoelastic materials is very weakly dependent on frequency [57]. This motivates the following constitutive relation in the frequency domain as an alternative to (2.8):

2.11 $$\sigma (\omega )=E(\omega )\left[\frac{1}{\left(1-2\nu \right)}e_{H}(\omega )+\frac{1}{\left(1+\nu \right)}e_{D}(\omega )\right].$$

In (2.11), only *one* of the material moduli depends on *ω*: the transform domain Young's modulus $E(\omega )=-i\omega E^{+}(\omega )$, with *E*^+^(*ω*) being the Fourier half-range transform of the extensional relaxation function $\hat{E}(t)$. Furthermore, we see that (2.11) is also in a hydrostatic/deviatoric split format. In the incompressible limit, κ→∞,ν→1/2 and together with $e_{H}\to 0$, (2.8) and (2.11) become, respectively,

2.12 $$\sigma (\omega )=-P(\omega )I+2M(\omega )e_{D}(\omega )$$

and

2.13 $$\sigma (\omega )=-P(\omega )I+\frac{2}{3}E(\omega )e_{D}(\omega ),$$

where P(ω) is the so-called *Lagrange multiplier* that accommodates the additional constraint of incompressibility. It is clear that for the consistency of (2.12) and (2.13) in the incompressible limit E(ω)=3M(ω), which is consistent given that E→3μ in this limit.

Traditionally, Prony series have been used extremely successfully to model the relaxation behaviour of a wide array of viscoelastic materials [58]. These usually take the form, e.g. for the extensional relaxation function

2.14 $$\hat{E}(t)=E_{0}+\sum_{r=1}^{N}E_{r}\;e^{-t/\tau_{j}},$$

where *τ*_*j*_ are the associated relaxation times of the medium in question. The simplest models accommodate a single relaxation time *τ* and a convenient choice of amplitudes gives

2.15 $$\hat{E}(t)=E^{\infty }+(E^{I}-E^{\infty })\;e^{-t/\tau },$$

where *E*^*I*^ and *E*^∞^ are known as the *instantaneous* and *long-term* Young's moduli and *τ* is the sole relaxation time of the material. In physical terms, *E*^*I*^ is Young's modulus that would be measured from the initial load curve when a material is subjected to rapid extension (on timescales *t*≪*τ*) up to a strain *e*_max_ and then held at this strain (termed a ‘stress relaxation test’). On the other hand, *E*^∞^ is Young's modulus that would be measured from the load curve when the medium is subjected to a very slow (quasi-static) extension on timescales *t*≫*τ*. The medium is perfectly elastic if *E*^*I*^ = *E*^∞^. The relaxation time *τ* is obtained by fitting the model to the relaxation test data [58]. We note that for QLV the relaxation functions can be obtained in the linear elastic regime since they are independent of deformation [40]. Employing the form (2.15), we find that

2.16 $$\begin{array}{rl} E(\omega ) & \;=-i\omega E^{+}(\omega ) \end{array}$$

2.17 $$\begin{array}{rl} & \;=E^{S}-iE^{L}, \end{array}$$

where

2.18 $$E^{S}=\frac{\left(E^{\infty }+E^{I}D^{2}\right)}{\left(1+D^{2}\right)}\;\mathrm{and}\;E^{L}=\frac{D(E^{I}-E^{\infty })}{\left(1+D^{2}\right)}.$$

In (2.18) we have introduced the *Deborah number D* = *ωτ* relating the relaxation time to the characteristic time of the deformation process (1/*ω*). Note that $E^{S}>0$ and $E^{L}>0$ represent the *storage* and *loss* moduli of the frequency domain viscoelastic Young's modulus E(ω), given that *E*^*I*^ > *E*^∞^. The latter inequality arises given that materials are ‘glassy’ (stiff) at high frequency and ‘rubbery’ (soft) at low frequency. The shear modulus can also be written in this form in the frequency domain, i.e. $M(\omega )=M^{S}-iM^{L}$.

## 3. Linear viscoelastic wave propagation through inhomogeneous rods

**Figure 1.**
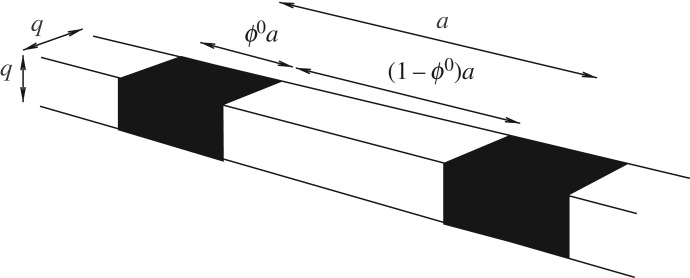
Illustrating the geometry of the periodic inhomogeneous rod. The periodic cell is of length *a*≫*q*, where *q* is the cross-sectional lengthscale of the rod. Phase 1 (black) has volume fraction *ϕ*^0^ and phase 2 (white) has volume fraction (1 − *ϕ*^0^).

Consider now viscoelastic wave propagation in a thin rod whose cross section has characteristic length scale *q* as depicted in figure 1. Pose the propagation of longitudinal elastic waves of the form

3.1 $$u=(u_{1}(x_{1}),u_{2}(x_{1},x_{3}),u_{3}(x_{1},x_{2}))\;e^{-i\omega t}$$

subject to lateral stress-free conditions *σ*_22_ = *σ*_33_ = 0 and with time-harmonic dependence ensuring that we work in the Fourier domain. Assume that *u*_1_(*x*_1_) = e^i*kx*_1_^ where *k* = 2*π*/λ is the wavenumber and λ is the wavelength and assume that *kq*≪1 which is the ‘thin rod’ regime. In this asymptotic regime, *u*_2_ ≈  − *νx*_2_*u*′_1_(*x*_1_), *u*_3_ ≈  − *νx*_3_*u*_1_′(*x*_1_) [59] and shear strains arising due to *u*_2,1_ and *u*_3,1_ (where we have defined the notation *u*_*i*,*j*_ = ∂*u*_*i*_/∂*x*_*j*_) are then an order of magnitude smaller than *u*_1_′(*x*_1_) given that *x*_1_ scales naturally on 1/*k*, whereas *x*_2_ and *x*_3_ scale on *q*. In the incompressible regime of interest here *ν* = 1/2. Under these conditions, *u*_1_(*x*_1_) satisfies the following governing equation in the frequency domain [60]:

3.2 $$\frac{\partial \;}{\partial x_{1}}\left(E(\omega )\frac{\partial u_{1}}{\partial x_{1}}\right)+\rho \omega^{2}u_{1}=0,$$

where *ρ* is the mass density. The displacement *u*_1_(*x*_1_) is the fundamental mode that would propagate in the rod at low frequencies, i.e. *kq*≪1 where $k=\rho \omega^{2}/E(\omega )$.

Suppose now that, although the rod has uniform cross-section, it is non-uniform in its mechanical properties, possessing a periodic microstructure with characteristic length scale of O(a). We assume that *q*≪*a* so that the rod can certainly be considered to be *thin* both with respect to the microstructure and the wavelength. Dispersive effects due to the thickness of the rod can therefore be considered to be negligible and longitudinal waves satisfy (3.2) now where E(ω) and *ρ* depend on *x*_1_ [60]. With reference to figure 1, we will consider a two phase, periodic composite rod consisting of a repeated periodic cell of length *a*, within which there are two isotropic, viscoelastic materials. Note that for ease of discussion we shall call phase 1 the *inclusion* material and denote its volume fraction in the material by *ϕ*^0^. In the following section, we will describe the influence of large, quasi-static pre-stress on viscoelastic wave propagation, while in this section we shall describe the situation when there is no pre-stress; this illustrates numerous aspects of viscoelastic wave propagation in heterogeneous media and sets the scene before we extend the study to incorporate the influence of pre-stress. While there has been significant focus on the propagation of viscoelastic waves in layered media [61,62] and the role of damping in viscoelastic phononic crystals [63–68], the regime of a thin inhomogeneous rod does not appear to have been discussed before. It therefore provides a convenient asymptotic regime in which to study the influence of viscoelasticity in the large deformation regime, as we will do in later sections once the un-stressed scenario has been considered here.

Referring to (3.2), the equation governing dimensional displacements *u*_1_(*x*_1_) in the inhomogeneous rod is

3.3 $$\frac{\partial }{\partial x}\left(E^{0}(x;\omega )\frac{\partial u_{1}}{\partial x_{1}}\right)+\omega^{2}\rho^{0}(x)u_{1}=0$$

where *ρ*^0^ is the inhomogeneous (and real) mass density in the unstressed state (hence the superposed 0), i.e.

3.4 $$\rho^{0}(x)=\left\{ \begin{array}{ll} \rho_{1}^{0}, & x\in a[n,n+\phi^{0}],\\ \rho_{2}^{0}, & x\in a[n+\phi^{0},n+1], \end{array}\right.$$

where n∈Z. Furthermore $E^{0}$ is the (inhomogeneous and complex) frequency domain Young's modulus in the unstressed state, i.e.

3.5 $$E^{0}(x;\omega )=\left\{ \begin{array}{ll} E_{1}^{0}(\omega )=E_{1}^{S0}(\omega )-iE_{1}^{L0}(\omega ), & x\in a[n,n+\phi^{0}],\\ E_{2}^{0}(\omega )=E_{2}^{S0}(\omega )-iE_{2}^{L0}(\omega ), & x\in a[n+\phi^{0},n+1]. \end{array}\right.$$

We assume that both materials can be described adequately by a single relaxation time (*τ*^1^ and *τ*^2^ in phases 1 and 2, respectively) Prony series of the form (2.15) and with associated instantaneous and long-term Young's moduli *E*^*I*^_1,2_, *E*^∞^_1,2_, respectively.

Define the non-dimensional variable $\tilde{x}=x/a$, and the scaled displacement $\tilde{u}(\tilde{x})=u(a\tilde{x})/U$ for some typical displacement magnitude *U*. Then the governing equation (3.3) can be written in the form

3.6 $$\frac{\partial }{\partial {\tilde{x}}_{1}}\left({\tilde{E}}^{0}({\tilde{x}}_{1};\tilde{D},{\tilde{\tau }}_{1},{\tilde{\tau }}_{2})\frac{\partial {\tilde{u}}_{1}}{\partial {\tilde{x}}_{1}}\right)+\epsilon^{2}{\tilde{\rho }}^{0}({\tilde{x}}_{1}){\tilde{u}}_{1}=0,$$

where ${\tilde{E}}^{0}(\tilde{x};\tilde{D},{\tilde{\tau }}_{1},{\tilde{\tau }}_{2})=E^{0}(x;\omega )/E_{c}$ with $\tilde{D}=\omega \tau_{c},{\tilde{\tau }}_{r}=\tau_{r}/\tau_{c}$, *r* = 1, 2 and ${\tilde{\rho }}^{0}(\tilde{x})=\rho^{0}(x)/\rho_{c}$. Furthermore, we have defined the non-dimensional parameter *ϵ* = *aω*/*c*_*c*_ = *k*_*c*_*a* where *c*_*c*_ = (*E*_*c*_/*ρ*_*c*_)^1/2^ is a low-frequency characteristic wave speed given that we have introduced the characteristic elastic Young's modulus, relaxation time and density *E*_*c*_, *τ*_*c*_ and *ρ*_*c*_ respectively. In particular, we note that the transform domain Young's modulus is piecewise constant, i.e.

3.7 $${\tilde{E}}^{0}(\tilde{x};\tilde{D},{\tilde{\tau }}_{1},{\tilde{\tau }}_{2})=\left\{ \begin{array}{ll} {\tilde{E}}_{1}^{0}(\tilde{D})={\tilde{E}}_{1}^{S}-i{\tilde{E}}_{1}^{L}, & \tilde{x}\in [n,n+\phi^{0}],\\ {\tilde{E}}_{2}^{0}(\tilde{D})={\tilde{E}}_{2}^{S}-i{\tilde{E}}_{2}^{L}, & \tilde{x}\in [n+\phi^{0},n+1], \end{array}\right.$$

where, with *r* = 1, 2,

3.8 $${\tilde{E}}_{r}^{S}={\tilde{E}}_{r}^{\infty }{\tilde{R}}_{r}^{S},\;{\tilde{E}}_{r}^{L}={\tilde{E}}_{r}^{\infty }{\tilde{R}}_{r}^{L}$$

and

3.9 $${\tilde{R}}_{r}^{S}=\frac{\left(1+{\tilde{E}}_{r}{\left(\tilde{D}{\tilde{\tau }}_{r}\right)}^{2}\right)}{\left(1+{\left(\tilde{D}{\tilde{\tau }}_{r}\right)}^{2}\right)},\;{\tilde{R}}_{r}^{L}=\frac{\tilde{D}{\tilde{\tau }}_{r}({\tilde{E}}_{r}-1)}{\left(1+{\left(\tilde{D}{\tilde{\tau }}_{r}\right)}^{2}\right)}$$

and where we have defined ${\tilde{E}}_{r}^{\infty }=E_{r}^{\infty }/E_{c},{\tilde{E}}_{r}^{I}=E_{r}^{I}/E_{c}$ and ${\tilde{E}}_{r}={\tilde{E}}_{r}^{I}/{\tilde{E}}_{r}^{\infty }>1$. Note that we require *k*_*c*_*q*≪1 in order for (3.6) to be valid; we are yet to choose *ϵ* = *k*_*c*_*a* but this is limited by the assumption *k*_*c*_*q*≪1, i.e. *ϵ* also cannot be ‘too large’, although provided *q*/*a* is small enough we can take *ϵ* as large as we wish. The scaled Young's modulus ${\tilde{E}}_{r}^{0},r=1,2$ depends on numerous parameters but we stress the dependence on $\tilde{D}$ since this is the dependence on the non-dimensional frequency parameter that generates a low-frequency dynamic response.

Let us now restrict attention to the case of main interest in this article: low-frequency propagation in the regime where frequency dependence can still arise from the viscoelastic behaviour of the phases that comprise the medium. When wavelengths are much longer than the microscale *a*, we are in the so-called *separation-of-scales* regime, so that *ϵ* = *k*_*c*_*a*≪1. Using asymptotic homogenization on (3.6), with the assumption that the parameters in the storage and loss moduli are all O(1), it is straightforward to show that [11,28] the effective Young's modulus in the unstressed state (scaled on *E*_*c*_) takes on the following *harmonic mean* form:

3.10 $${\tilde{E}}_{*}^{0}(\tilde{D})={\left(\frac{\phi^{0}}{{\tilde{E}}_{1}^{0}(\tilde{D})}+\frac{\left(1-\phi^{0}\right)}{{\tilde{E}}_{2}^{0}(\tilde{D})}\right)}^{-1}=\frac{{\tilde{E}}_{1}^{0}(\tilde{D}){\tilde{E}}_{2}^{0}(\tilde{D})}{\left(1-\phi^{0}){\tilde{E}}_{1}^{0}(\tilde{D})+\phi^{0}{\tilde{E}}_{2}^{0}(\tilde{D}\right)}.$$

Recall once again that ${\tilde{E}}_{1}^{0}$ and ${\tilde{E}}_{2}^{0}$ depend on the non-dimensional frequency $\tilde{D}$ even for *ϵ*≪1. This dependence on *ω* is solely due to the presence of the (re-scaled) Deborah number $\tilde{D}=\omega \tau_{c}$ in the viscoelastic storage and loss moduli. The effective modulus will, of course, also depend on the relaxation time parameters ${\tilde{\tau }}_{r}$ but as noted above we explicitly note its dependence on $\tilde{D}$ only due to our interest on low-frequency dispersive behaviour. What this means is that even for *ϵ*≪1, where in the purely elastic case we would be in the homogenization regime with a constant effective Young's modulus, we now have a frequency-dependent effective Young's modulus ${\tilde{E}}_{*}^{0}={\tilde{E}}_{*}^{0}(\tilde{D})$, even in this homogenization regime.

With phase behaviour as described above, the effective (complex) Young's modulus (3.10) is therefore written as

3.11 $${\tilde{E}}_{*}^{0}(\tilde{D})={\tilde{E}}_{*}^{S0}(\tilde{D})-i{\tilde{E}}_{*}^{L0}(\tilde{D}).$$

Given that this is the frequency (Fourier) domain effective modulus, the time domain relaxation function associated with extensional deformation can be obtained by Fourier inverting (3.11). This is of practical interest, because it is clear that even in this simple geometry the relaxation function of an inhomogeneous medium is not a simple linear superposition of the relaxation functions of its constituents. Since here we are primarily interested in time-harmonic wave propagation problems, we are mainly interested in the frequency domain response.

**Figure 2.**
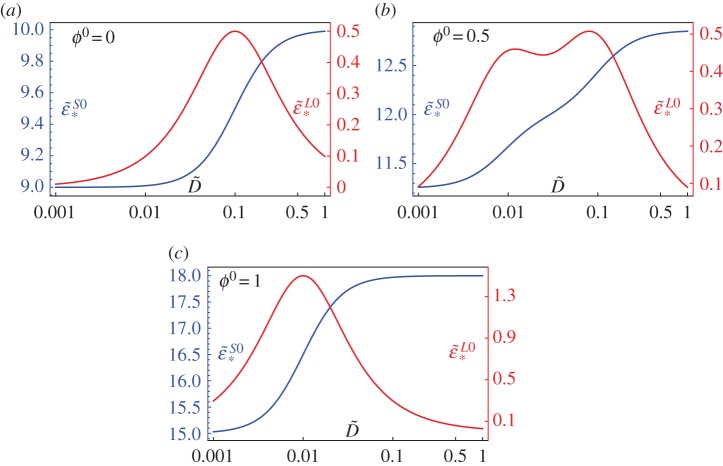
Illustrating the effective storage and loss modulus of a composite rod with volume fraction (*a*) *ϕ*^0^ = 0 (pure host), (*b*) *ϕ*^0^ = 0.5, (*c*) *ϕ*^0^ = 1 (pure inclusion) and parameters defined in (3.12). Note in particular the broader band attenuation in (*b*) accompanied by a less extreme glass transition region. (Online version in colour.)

In figure 2, we illustrate the effective response by plotting the effective storage and loss moduli for the specific example when

3.12 $${\tilde{E}}_{1}^{\infty }=15,\;{\tilde{E}}_{1}^{I}=18,\;{\tilde{\tau }}_{1}=100,\;{\tilde{E}}_{2}^{\infty }=9,\;{\tilde{E}}_{2}^{I}=10\;\mathrm{and}\;{\tilde{\tau }}_{2}=10$$

where we have taken *E*_*c*_ = 10^5^ Pa, *τ*_*c*_ = 1*s*, given that these properties are typical of polyurethanes when varying composition and cross-linked densities [69,70], noting in particular that relaxation times for polyurethane can vary between 10 and 1000*s*. In particular, given that $\tilde{D}=\omega \tau_{c}=\omega$, it is clear that there is a strong frequency dependence even in the separation of scales (homogenization) regime. Note the blending of loss modulus amplitudes, and therefore a broader band attenuative effect at low frequency with the two phase medium at *ϕ*^0^ = 0.5 in figure 2*b*. The frequency at which the storage modulus increases (usually significantly) is usually termed the *glass transition region*, referring to the associated increase in stiffness.

## 4. Quasi-static deformation of a quasi-linear viscoelastic medium

Note that with the configuration considered above, the effective properties of the periodic rod are fixed once the material properties of the constituent phases and their volume fractions are specified. One can envisage many situations where one would like to modify the effective properties of a material *in situ*. As discussed in the Introduction, over the last decade a significant amount of research has been conducted into the use of nonlinear elastic materials as a means to modify the incremental dynamic response of a medium. Here we study the influence of viscoelastic effects on longitudinal wave propagation in thin rods when the rod is subject to uniaxial deformation via a tensile stress *T*. As we shall describe in §4c, we shall assume that the initial large deformation of the thin rod is *quasi-static* and piecewise *homogeneous*. Strictly, given that the rod is inhomogeneous, complex local deformations would result close to interfaces. However, these will be confined to small regions within the vicinity of the interfaces and would certainly not have a dominant response in the low-frequency regime of interest here. Before analysing the structure of the inhomogeneous rod let us consider what it means to be in the quasi-static regime of a viscoelastic material that has been subjected to finite deformation. For convenience, we do not append a hat to time varying variables in this section as we have done above, because we are not envisaging taking Fourier transforms of these variables - they are associated with the large deformation problem.

### (a) Quasi-static deformations

We shall study nonlinear viscoelastic media that behave in a manner described by the *QLV theory* [40,45,71]. The general constitutive expression for anisotropic media takes the form

4.1 $$\pi^{\mathrm{ve}}(t)=\int_{-\infty }^{t}G(t-s):\frac{\partial \pi^{e}(s)}{\partial s}\;ds,$$

where **π** is the second Piola–Kirchhoff stress with superscripts ‘ve’ and ‘e’ referring to the *viscoelastic* and *elastic* stresses, respectively, and **G** is a fourth-order *reduced* relaxation tensor, where *reduced* refers to the fact that it is non-dimensional, unlike the relaxation tensor in linear viscoelasticity, which has dimensions of stress, see e.g. (2.3). The stress **π**^e^ is the (instantaneous) *elastic* stress, derived from a strain energy *W* as is standard in nonlinear elasticity. The form prescribed in (4.1) preserves objectivity and provides a balance between realistic modelling and ease of implementation in computational simulations. Of specific note and of importance in what is to follow in this article, for isotropic, incompressible QLV materials, *relaxation is independent of strain*. Recall that the Cauchy stress **T** is related to **π** by

4.2 $$\pi =JF^{-1}TF^{-T}$$

where **F** is the deformation gradient while the superscript ‘T’ denotes its transpose and *J* = det**F**. Restricting attention now to the scenario of interest in the present paper, isotropic incompressible media, equation (4.1) takes the form [40]

4.3 $$T^{\mathrm{ve}}(t)=-P(t)I+F(t)\left(D(0)\pi_{D}^{e}(t)+\int_{0}^{t}D^{\prime }(t-s)\pi_{D}^{e}(s)\;ds\right)F^{T}(t)$$

where *P*(*t*) is the Lagrange multiplier associated with satisfying the constraint of incompressibility. D(t) is the reduced deviatoric scalar relaxation function, which without loss of generality is defined subject to the condition that D(0)=1 and furthermore

4.4 $$\pi_{D}^{e}(t)=F^{-1}T_{D}^{e}F^{-T}=2\left[\left(\frac{I_{2}}{3}W_{2}-\frac{I_{1}}{3}W_{1}\right)C^{-1}+W_{1}I-W_{2}C^{-2}\right].$$

It should be noted that

4.5 $$T_{D}^{e}=T^{e}-\frac{1}{3}\;\mathrm{tr}(T^{e})I$$

is the deviatoric part of the Cauchy stress. **C** = **F**^T^**F** is the right Cauchy–Green strain tensor and *W*_*i*_, (*i* = 1, 2) is the derivative of the strain energy function *W* with respect to the invariants *I*_*i*_, (*i* = 1, 2) of **C**,

4.6 $$I_{1}=\mathrm{tr}C,\;I_{2}=\frac{1}{2}[{\left(\mathrm{tr}C\right)}^{2}-\mathrm{tr}C^{2}]=(\det C)\;\mathrm{tr}(C^{-1}),$$

and furthermore $I_{3}=J^{2}=\det C=1$ due to the constraint of incompressibility.

In a homogeneous, isotropic and incompressible material subjected to a simple uniaxial homogeneous extension in the uniaxial direction *x*_1_, a point in the undeformed configuration, prescribed by Cartesian coordinates (*X*_1_, *X*_2_, *X*_3_) moves to

4.7 $$x_{1}(t)=\lambda (t)X_{1},\;x_{2}(t)=\frac{1}{\sqrt{\lambda (t)}}X_{2}\;\mathrm{and}\;x_{3}(t)=\frac{1}{\sqrt{\lambda (t)}}X_{3},$$

in the deformed configuration, where λ is the uniaxial stretch along the *x*_1_-direction. Here we suppose that such a deformation has arisen given the uniaxial stress distribution of the form

4.8 $$T_{11}(t)=T(t),\;T_{22}(t)=T_{33}(t)=0\;\mathrm{and}\;T_{ij}=0\;(i\neq j),$$

where *T*(*t*) is therefore the uniaxial tensile load. Under the quasi-linear viscoelastic stress–strain law (4.3), it is straightforward now to determine the relationship between *T*(*t*) and λ(*t*), starting by writing down the expressions for the diagonal stresses, i.e.

4.9 $$T_{11}^{\mathrm{ve}}(t)=T(t)=\lambda^{2}(t)\left(\Pi_{D11}^{e}(t)+\int_{0}^{t}D^{\prime }(t-s)\Pi_{D11}^{e}(s)\;ds\right)-P(t)$$

and

4.10 $$T_{22}^{\mathrm{ve}}(t)=T_{33}^{\mathrm{ve}}(t)=0=\lambda^{-1}(t)\left(\Pi_{D22}^{e}(t)+\int_{0}^{t}D^{\prime }(t-s)\Pi_{D22}^{e}(s)\;ds\right)-P(t).$$

Thus, by substracting (4.10) from (4.9), we obtain

4.11 $$\begin{array}{r} T(t)=\lambda^{2}(t)\Pi_{D11}^{e}(t)-\frac{1}{\lambda (t)}\Pi_{D22}^{e}(t)+\int_{0}^{t}D^{\prime }(t-s)\left(\lambda^{2}(t)\Pi_{D11}^{e}(s)-\frac{1}{\lambda (t)}\Pi_{D22}^{e}(s)\right)ds, \end{array}$$

where from (4.4)

4.12 $$\Pi_{D11}^{e}=2\left[\frac{2}{3}\left(W_{1}+\frac{W_{2}}{\lambda }\right)\left(1-\frac{1}{\lambda^{3}}\right)\right]$$

and

4.13 $$\Pi_{D22}^{e}=2\left[\frac{1}{3}\left(W_{1}+\frac{W_{2}}{\lambda }\right)\left(1-\lambda^{3}\right)\right].$$

At this point, let us be more specific about the type of material under study. We consider *Mooney–Rivlin* materials, with strain energy function of the form [72]

4.14 $$W=\frac{\mu^{I}}{2}\left(\frac{1}{2}+\gamma \right)(I_{1}-3)+\frac{\mu^{I}}{2}\left(\frac{1}{2}-\gamma \right)(I_{2}-3),$$

where *γ* is a constant in the range −1/2 ≤ *γ* ≤ 1/2 and *μ*^*I*^ is the infinitesimal instantaneous shear modulus. For the reduced relaxation function, we consider the classical one-term Prony series form [58]

4.15 $$D(t)=\frac{\mu^{\infty }}{\mu^{I}}+\left(1-\frac{\mu^{\infty }}{\mu^{I}}\right)e^{-t/\tau },$$

where *μ*^∞^ is the *long-term* shear modulus and *τ* denotes the relaxation time. The scaling used in the reduced relaxation function with *μ*^*I*^ factored out in order to appear in the strain energy function (4.14), ensures that D(0)=1. However, if one wished, *μ*^∞^ could be employed in the strain energy function in place of *μ*^*I*^. This would yield a different scaling of the reduced relaxation function, leading to $D(0)=\mu^{I}/\mu^{\infty }$, which would then be a factor appearing in (4.3).

Finally, equation (4.11) becomes

4.16 $$\begin{array}{rl} \tilde{T}(t) & \;=\frac{T(t)}{\mu^{I}}=\ell (s)\lambda (t)\left(1-\frac{1}{\lambda^{3}(t)}\right)\\ & \;\;+\frac{1}{3\tau }\int_{0}^{t}\left(\frac{\mu^{\infty }}{\mu^{I}}-1\right)e^{-\left(t-s\right)/\tau }\ell (s)\left(\frac{2\lambda^{2}(t)}{\lambda (s)}+\frac{\lambda^{2}(s)}{\lambda (t)}\right)\left(1-\frac{1}{\lambda^{3}(s)}\right)ds, \end{array}$$

where

4.17 $$\ell (t)=\left(\frac{1}{2}-\gamma \right)+\lambda (t)\left(\frac{1}{2}+\gamma \right).$$

### (b) Relaxation and creep tests: the equivalence of the quasi-static limit withhyperelastic theory

We now describe how the large time limit of the QLV theory, which we shall term as the *quasi-static limit*, is equivalent to a purely hyperelastic deformation having the same strain energy function as that used in the QLV analysis but with the *μ*^*I*^ in (4.14) interchanged with *μ*^∞^ given that this is associated with long-term deformation. Note that one can impose the stretch λ(*t*) and determine the resulting time-dependent stress *T*(*t*) or vice-versa. The latter is more challenging mathematically given that (4.16) is a nonlinear integral equation for the unknown λ(*t*), although an efficient method to determine the solution of this equation was introduced in [40]. As an example of the resulting deformations, suppose that the relaxation time of a homogeneous material is *τ* = 2 *s* and therefore define *τ*_*c*_ = *τ* and $\tilde{t}=t/\tau$. We can consider imposing either the deformation in the ‘ramp’ form

**Figure 3.**
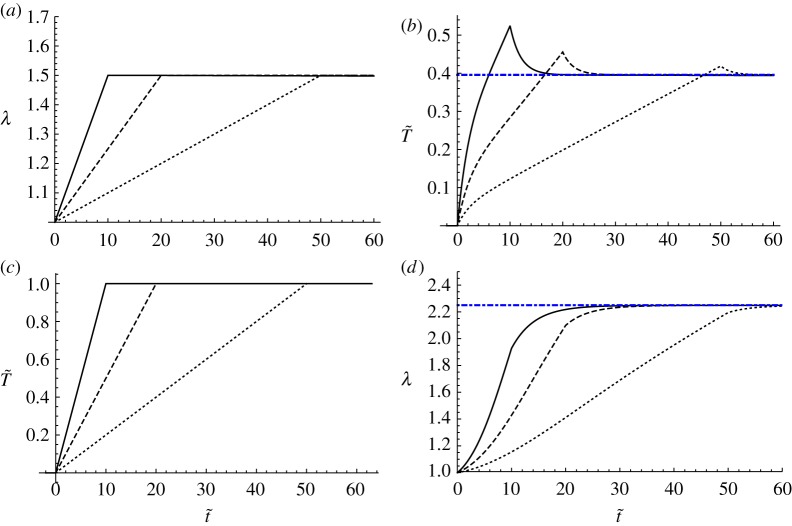
Relaxation and creep curves associated with uniaxial tension of a quasi-linear viscoelastic Mooney–Rivlin medium with relaxation time *τ* = *τ*_*c*_ = 2*s* and *μ*^∞^/*μ*^*I*^ = 0.3. (*a*) Imposed stretch history as in (4.18) with subsequent predictions in (*b*) of $\tilde{T}=T/\mu^{I}$. (*c*) Imposed stress history as in (4.19) with subsequent predictions (*d*) of λ. In both (*a*) and (*c*), three different rates were considered, $\bar{t}=10$ (solid), $\bar{t}=20$ (dashed), $\bar{t}=50$ (dotted) noting that both $\tilde{t}$ and $\bar{t}$ are scaled on *τ*. The blue dot-dashed straight line in (*b*) and (*d*) indicates the quasi-static limit as determined by hyperelastic theory using the same strain energy function as in QLV, but employing the long-term shear modulus *μ*^∞^. (Online version in colour.)

4.18 $$\lambda (t)=\left\{ \begin{array}{ll} 1.5\tilde{t}, & 0\leq \tilde{t}\leq \bar{t},\\ 1.5, & t\geq \bar{t}, \end{array}\right.$$

as depicted in figure 3*a* or we can pose the non-dimensional tension $\tilde{T}=T/\mu^{I}$ in the ramp form

4.19 $$\tilde{T}(t)=\left\{ \begin{array}{ll} \tilde{t}, & 0\leq \tilde{t}\leq \bar{t},\\ \bar{t}, & \tilde{t}\geq \bar{t}, \end{array}\right.$$

as depicted in figure 3*c*. Imposing stretch as in (4.18) gives rise to the classical relaxation test, with the resulting tension predicted in figure 3*b* for three different rates $\bar{t}=10,20,50$, noting that this is a time scaled on *τ*. In this instance, the tension follows directly by computing the integrals on the right-hand-side of (4.16) at successive times. Of particular note is that the slower the deformation the smaller the relaxation effect, although regardless of this, $\tilde{T}$ tends to a fixed value as $\tilde{t}\to \infty$. Analogously in figure 3*d*, the respective stretch predictions are plotted when the ramp stress (4.19) is imposed with $\bar{t}=10,20,50$, by solving the integral equation that results from (4.16) as described in [40]. In all plots here a Mooney–Rivlin strain energy function is taken with *γ* = 0 and we also took *μ*^∞^/*μ*^*I*^ = 0.3.

We note that the horizontal ‘dot-dashed’ lines in figure 3*b*,*d* are the long-term viscoelastic deformations and can be determined from standard hyperelastic theory via the relations (see e.g. ch. 1 of [7])

4.20 $$T=-PI+\beta_{1}B+\beta_{2}B^{2},$$

where **B** = **FF**^T^ and

4.21 $$\beta_{1}=2(W_{1}+I_{1}W_{2})\;\mathrm{and}\;\beta_{2}=-2W_{2},$$

noting that for the calculation leading to the horizontal dot-dashed lines in figures 3*b*,*d*, the Mooney–Rivlin strain energy function (4.14) has been employed, but with *μ*^*I*^ interchanged with *μ*^∞^, i.e.

4.22 $$W_{\mathrm{MR}}=\frac{\mu^{\infty }}{2}\left(\frac{1}{2}+\gamma \right)(I_{1}-3)+\frac{\mu^{\infty }}{2}\left(\frac{1}{2}-\gamma \right)(I_{2}-3),$$

where here, *γ* = 1/2 recovers the neo-Hookean model *W*_NH_. This interchange of moduli in the case of hyperelasticity is consistent given that we wish to determine the long-term viscoelastic deformation, and its accuracy is confirmed by the agreement of the long-time QLV and hyperelastic results depicted in figure 3*b*,*d*. In particular, for the uniaxial elastic deformation described by

4.23 $$\lambda_{1}=\lambda \;\mathrm{and}\;\lambda_{2}=\lambda_{3}=\frac{1}{\sqrt{\lambda }}$$

relations (4.20) give

4.24 $$T=-P+\beta_{1}\lambda^{2}+\beta_{2}\lambda^{4}\;\mathrm{and}\;0=-P+\frac{\beta_{1}}{\lambda }+\frac{\beta_{2}}{\lambda^{2}}$$

so that

4.25 $$T(\lambda )=\beta_{1}\left(\lambda^{2}-\frac{1}{\lambda }\right)+\beta_{2}\left(\lambda^{4}-\frac{1}{\lambda^{2}}\right),$$

for a general strain energy function. For the specific case of a Mooney–Rivlin strain energy this becomes

4.26 $$T(\lambda )=\mu^{\infty }\left[\left(\frac{1}{2}+\gamma \right)\left(\lambda^{2}-\frac{1}{\lambda }\right)+\left(\frac{1}{2}-\gamma \right)\left(\lambda -\frac{1}{\lambda^{2}}\right)\right].$$

For the scenario depicted in figure 3*b* when *γ* = 0, the long-term elastic limit is

4.27 $$\tilde{T}=\frac{T}{\mu^{I}}=\frac{\mu^{\infty }}{2\mu^{I}}\left(\lambda^{2}+\lambda -\frac{1}{\lambda }-\frac{1}{\lambda^{2}}\right).$$

For more details on the problem of simple uniaxial extension, we refer to ch. 3 of [73] (in particular see §§3.1.1 and 3.2.1).

### (c) Quasi-static pre-stress of an inhomogeneous incompressible rod

We now consider the nonlinear deformation of an inhomogeneous incompressible rod which has periodic microstructure as in §3. We assume that each phase of the rod is quasi-linear viscoelastic but is subjected to a slow ramp deformation and we are interested only in the large time limit, which as we have shown above is equivalent to the hyperelastic theory with an appropriate strain energy function. In the deformed state, the length and width of the periodic cell of the rod will change to *a*′ and *q*′, respectively (deformed from *a* and *q* in the unstressed state) and hence if we non-dimensionalize these on *a*′ we find that in the deformed configuration, the *n*th periodic cell is defined by the domain

4.28 $$D_{n}=\left\{n\leq {\tilde{x}}_{1}\leq n+1,-\frac{\eta^{\prime }}{2}\leq {\tilde{x}}_{2},{\tilde{x}}_{3}\leq \frac{\eta^{\prime }}{2}\right\},$$

where *η*′ = *q*′/*a*′≪1 and noting that we have used a tilde on spatial variables given that we have non-dimensionalized.

The inhomogeneous rod will be subjected to uniaxial tension as in (4.8) with *T*_11_ = *T* and lateral stress-free conditions. Since the cell is long and thin (*η*′≪1), we neglect the effects of *necking* close to the phase interfaces and assume a simple planar deformation where each cross-sectional ${\tilde{x}}_{2}{\tilde{x}}_{3}$ plane within each phase deforms identically. This appears to be a reasonable approximation for *η*′≪1 and will certainly capture the leading-order behaviour. This assumption is similar to the approximation made in [37,74,75], for example (although the nature of the approximation is not discussed there). As a result of these assumptions together with the constraint of incompressibility, the resulting deformation in the *r*th phase (*r* = 1, 2) is described by

4.29 $${\tilde{x}}_{1}=\Lambda_{r}{\tilde{X}}_{1}+\gamma_{r}^{\left(n\right)},\;{\tilde{x}}_{2}=\frac{{\tilde{X}}_{2}}{\sqrt{\Lambda_{r}}}\;\mathrm{and}\;{\tilde{x}}_{3}=\frac{{\tilde{X}}_{3}}{\sqrt{\Lambda_{r}}},$$

where *Λ*_*r*_ and $1/\sqrt{\Lambda_{r}}$ are the principal stretches in the longitudinal and lateral directions in the *r*th phase, *r* = 1, 2. The constants *γ*^(*n*)^_*r*_ are associated with a rigid body motion in the *r*th phase and *n*th cell. Such rigid body displacements are required in order to satisfy the continuity of displacement boundary conditions on the interfaces between the phases, as will be described below.

Given continuity of tension across phase interfaces and assuming *T* is imposed, the stress–stretch relations for each phase, derived from (4.25), yield an equation for the principle stretch *Λ*_*r*_ in phase *r* = 1, 2. The resulting Lagrangian forms of displacements are

4.30 $$\begin{array}{rl} U_{1r}^{\left(n\right)} & \;={\tilde{x}}_{1}-{\tilde{X}}_{1}=(\Lambda_{r}-1){\tilde{X}}_{1}+\gamma_{r}^{\left(n\right)}, \end{array}$$

4.31 $$\begin{array}{rl} U_{2r}^{\left(n\right)} & \;={\tilde{x}}_{2}-{\tilde{X}}_{2}=\left(\frac{1}{\sqrt{\Lambda_{r}}}-1\right){\tilde{X}}_{2} \end{array}$$

4.32 $$\begin{array}{rl} \mathrm{and}\;\;U_{3r}^{\left(n\right)} & \;={\tilde{x}}_{3}-{\tilde{X}}_{3}=\left(\frac{1}{\sqrt{\Lambda_{r}}}-1\right){\tilde{X}}_{3}, \end{array}$$

where as noted above, the constants *γ*^(*n*)^_*r*_ are deduced by insisting on continuity of displacements at ${\tilde{X}}_{1}=n,n+\phi^{0}$, i.e.

4.33 $$(\Lambda_{1}-1)(n+\phi^{0})+\gamma_{1}^{\left(n\right)}=(\Lambda_{2}-1)(n+\phi^{0})+\gamma_{2}^{\left(n\right)}$$

and

4.34 $$(\Lambda_{2}-1)n+\gamma_{2}^{\left(n-1\right)}=(\Lambda_{1}-1)n+\gamma_{1}^{\left(n\right)}.$$

These are coupled with the assumption that (without loss of generality) *γ*^(0)^_2_ = 0, which gives a fixed reference. We note again that the specific values of *γ*^(*n*)^_*r*_ are merely rigid body displacements required in order to satisfy the continuity of the body. The nonlinear deformation above now serves as an equilibrium state from which to perturb via the consideration of superposed longitudinal waves.

## 5. Longitudinal waves in pre-stressed elastic and quasi-linear viscoelastic thin rods

Before considering wave propagation in the pre-stressed *inhomogeneous* medium, let us consider how one determines the incremental equations in a homogeneous quasi-linear viscoelastic rod. We first summarize the existing theory for hyperelastic rods before deriving the theory in the QLV scenario.

### (a) Incremental elastic waves

The classical theory of ‘small-on-large’ allows one to write down the equations governing small-amplitude elastic vibrations superposed on nonlinear elastic deformation and here we briefly summarize the theory for the case of hyperelastic materials [7]. In the context of a *homogeneous* incompressible thin rod, consider an initial uniaxial deformation as in (4.23) and (4.24), before assuming the presence of a superposed longitudinal wave of the form (3.1), working in the same asymptotic regime as that following (3.2) so that we neglect shear strains and lateral displacements are such that

5.1 $$u_{2,2}=u_{3,3}=-\nu u_{1,1}=-\frac{1}{2}u_{1,1}$$

for the incompressible case studied here. Assuming that the incremental longitudinal stress is *Σ* we therefore have the incremental relations (see e.g. (3.16) in ch. 1 of [7])

5.2 $$\begin{array}{rl} \Sigma & \;=\left(A_{11}+P\right)u_{1,1}+A_{12}u_{2,2}+A_{13}u_{3,3}-p, \end{array}$$

5.3 $$\begin{array}{rl} 0 & \;=A_{21}u_{1,1}+\left(A_{22}+P\right)u_{2,2}+A_{23}u_{3,3}-p \end{array}$$

5.4 $$\begin{array}{rl} \mathrm{and}\;0 & \;=A_{31}u_{1,1}+A_{32}u_{2,2}+\left(A_{33}+P\right)u_{3,3}-p, \end{array}$$

where *P* is the Lagrange multiplier derived from (4.24) and *p* is the incremental Lagrange multiplier. The incremental moduli $A_{ij}$ are defined as

5.5 $$A_{ij}=\lambda_{i}\lambda_{j}\frac{\partial^{2}W}{\partial \lambda_{i}\partial \lambda_{j}},\;\left(\mathrm{no sum over}\;i,j\right)$$

noting that $A_{ij}=A_{ji}$ and due to symmetry $A_{21}=A_{31},A_{22}=A_{33}$. The relations (5.1) give

5.6 $$p=\left[A_{21}-\frac{1}{2}\left(A_{22}+A_{23}+P\right)\right]u_{1,1}$$

and the incremental stress–strain relationship is then given by

5.7 $$\Sigma =Au_{1,1},$$

where

5.8 $$A=\left[A_{11}-A_{21}-\frac{1}{2}\left(A_{12}+A_{13}-A_{22}-A_{23}\right)+\frac{3}{2}P\right].$$

The resulting equation governing *u*_1_(*x*_1_) is therefore

5.9 $$A\frac{\partial^{2}u_{1}}{\partial {x_{1}}^{2}}+\rho \omega^{2}u_{1}=0$$

where *ρ* = *ρ*^0^ given that the medium is incompressible. For the specific Mooney–Rivlin strain energy function, we find that

5.10 $$A=\mu^{\infty }\left(\frac{1}{2}+\gamma \right)\left(\lambda^{2}+\frac{2}{\lambda }\right)+\mu^{\infty }\left(\frac{1}{2}-\gamma \right)\frac{3}{\lambda^{2}}$$

and we note that this agrees with the form deduced in the same asymptotic (thin rod) limit in (3.10) of [76]. When $\lambda =1,A=3\mu^{\infty }=E^{\infty }$.

### (b) Incremental viscoelastic waves

Although the small-on-large theory associated with hyperelastic materials is well established, the analogous theory associated with QLV materials is not. We shall therefore derive such a theory, tailored to the specific configuration of interest here. A more general theory shall be developed elsewhere in the future. In addition to the large time-dependent uniaxial deformation (4.7), let us consider a separate deformation that is ‘close’ to the deformation (4.7), denoted by $\bar{x}=\chi (X)$. We define the difference between position vectors in the deformed configurations as

5.11 $$u=\bar{x}-x.$$

The total deformation gradient can be written as

5.12 $$\bar{F}=\mathrm{Grad}\;\bar{x}=F+\gamma F$$

where ***γ*** = grad ***u*** and Grad and grad denote the gradient operator with respect to **X** and **x**, respectively. Furthermore, $\bar{J}=\det\;\bar{F}=J+\mathrm{tr}(\gamma )J$ to first order in the perturbation and hence incompressibility requires tr(***γ***) = *u*_*k*,*k*_ = 0 as expected. Let us write the total Cauchy stress $\bar{T}$ and Piola–Kirchhoff stress $\bar{\pi }$ in the form

5.13 $$\bar{T}=T+\tau \;\mathrm{and}\;\bar{\pi }=\pi +\pi$$

so that ***τ*** and ***π*** denote the difference in Cauchy and second Piola–Kirchhoff stresses between the two deformation states. From (4.2),

5.14 $$\pi =F^{-1}TF^{-T}\;\mathrm{and}\;\pi =F^{-1}\left(\tau -\gamma T-T\gamma^{T}\right)F^{-T}.$$

The total viscoelastic Cauchy stress can therefore be written as

5.15 $${\bar{T}}^{\mathrm{ve}}=T^{\mathrm{ve}}+\tau^{\mathrm{ve}},$$

where

5.16 $$T^{\mathrm{ve}}=-PI+F\left(\pi_{D}^{e}+\int_{0}^{t}D^{\prime }(t-s)\pi_{D}^{e}(s)\;ds\right)F^{T}$$

and

5.17 $$\tau^{\mathrm{ve}}=-pI+\gamma_{1}\pi_{D}^{e}+F\pi_{D}^{e}F^{T}+\pi_{D}^{e}\gamma^{T}+\gamma T^{\mathrm{ve}}+F\left(\int_{0}^{t}D^{\prime }(t-s)\pi_{D}^{e}(s)\;ds\right)F^{T}+T^{\mathrm{ve}}\gamma^{T},$$

where *p* is the incremental lagrange multiplier such that $\bar{P}=P+p$, and is determined by imposing *τ*^ve^_22_ = 0. Note that

5.18 $$\pi_{D}^{e}=F^{-1}T_{D}^{e}F^{-T}\;\mathrm{and}\;\pi_{D}^{e}=F^{-1}\left(\tau_{D}^{e}-\gamma T_{D}^{e}-T_{D}^{e}\gamma^{T}\right)F^{-T}.$$

Finally, it should be noted that the leading-order stress defined in (5.16) is purely time-dependent, whereas the perturbation defined in (5.17) is both time *and* space-dependent.

Next, denoting the divergence operator with respect to ***x*** ($\bar{x}$) by div ($\bar{\mathrm{div}}$) and neglecting inertia associated with the initial finite deformation, the conservation of momentum equation takes the form

5.19 $$\bar{\mathrm{div}}\;{\bar{T}}^{\mathrm{ve}}=\rho \ddot{u},$$

where *ρ* = *ρ*^0^, recalling that *ρ*^0^ is the mass per unit of volume in the undeformed configuration. At leading order, (5.19) is

5.20 $$\mathrm{div}\;T^{\mathrm{ve}}=0,$$

whilst upon defining ***Σ***^ve^ = ***τ***^ve^ − ***γ*T**^ve^, the equation governing the perturbation is

5.21 $$\mathrm{div}\;\Sigma^{\mathrm{ve}}=\rho \ddot{u}.$$

Assume that the incremental displacement field takes the form (consistent with the longitudinal waves described above)

5.22 $$u(x_{1},x_{2},x_{3},t)=\left(u_{1}(x_{1}),u_{2}(x_{1},x_{2}),u_{3}(x_{1},x_{3})\right)H(t-t_{1})\;e^{-i\omega t},$$

where H denotes the Heaviside function, indicating that this perturbation is ‘switched on’ at time *t* = *t*_1_, where *t*_1_≫0 and

5.23 $$\gamma (x,t)=e^{-i\omega t}\left(\begin{array}{ccc} u_{1,1} & 0 & 0\\ u_{2,1} & -\frac{1}{2}u_{1,1} & 0\\ u_{3,1} & 0 & -\frac{1}{2}u_{1,1} \end{array}\right).$$

Now consider the QLV theory for the specific case of a Mooney–Rivlin material defined by (4.14) and for the uniaxial deformation defined in (4.7). It can be shown that the resulting incremental viscoelastic stresses take the form

5.24 $$\Sigma_{11}^{\mathrm{ve}}(t)/\mu^{I}=\left(f(t)+\int_{t_{1}}^{t}D^{\prime }(t-s)g(s,t)\;ds\right)e^{-i\omega t}u_{1,1}+\int_{t_{1}}^{t}D^{\prime }(t-s)h(s,t)\;e^{-i\omega s}u_{1,1}\;ds$$

and

5.25 Σ21ve(t)/μI =Σ31ve(t)/μI=ℓ(t)λ2(t) e−iωtu2,1 +13λ(t)∫t1tD′(t−s)ℓ(s)λ5/2(s)((2+λ3(s))λ3/2(t) e−iωs−λ3/2(s)(λ3(s)−1) e−iωtℓ(s)λ5/2(s)) u2,1ds

and the shear stresses are therefore set to zero upon neglecting shear strains. The contribution to (5.21) is then only due to *Σ*^ve^_11_ so that the equation of motion reduces to

5.26 $$\left(f(t)+\int_{t_{1}}^{t}D^{\prime }(t-s)(g(s,t)+h(s,t)\;e^{i\omega (t-s)})\;ds\right)u_{1,11}=-\frac{\rho \omega^{2}}{\mu^{I}}u_{1}$$

where

5.27 $$\begin{array}{rl} f(t) & \;=\frac{1}{\lambda^{2}(t)}\left[3\left(\frac{1}{2}-\gamma \right)+\left(\frac{1}{2}+\gamma \right)\lambda (t)\left(\lambda^{3}(t)+2\right)\right], \end{array}$$

5.28 $$\begin{array}{rl} g(s,t) & \;=\frac{2\left(1-\lambda^{3}(s)\right)}{3\lambda^{4}(s)\lambda (t)}[\ell (s)\left(\lambda^{3}(s)-\;\lambda^{3}(t)\right)], \end{array}$$

5.29 $$\begin{array}{rl} h(s,t) & \;=\frac{1}{6\lambda^{4}(s)\lambda (t)}[\lambda^{3}(s)\left(1-2\gamma +\lambda^{3}(s)(2(1-2\gamma )+3(1+2\gamma )\lambda (s))\right) \end{array}$$

5.30 $$\begin{array}{rl} & \;\;+2\lambda^{3}(t)\left(4-8\gamma +(2\gamma -1)\lambda^{3}(s)+(6\gamma +3)\lambda (s)\right)]. \end{array}$$

Now assume that $t_{1}\gg \bar{t}$ so that the initial ramp deformation has reached a steady-state (see §4b) and therefore λ(*s*) appearing in the integrand of (5.26) becomes independent of *s* for the times in the domain of integration. Further, consider *t*≫*t*_1_ and *t*≫*τ*, so that any transients on the left-hand side of (5.26) decay to zero. In this quasi-static regime, the equation governing perturbations is then

5.31 $$A(\lambda )\left(R^{S}(\omega )-iR^{L}(\omega )\right)\frac{\partial^{2}u_{1}}{\partial {x_{1}}^{2}}=-\rho \omega^{2}u_{1},$$

where A is as defined for a Mooney–Rivlin material in (5.10) and we have also defined

5.32 $$R^{S}(\omega )=\frac{1+\tilde{E}D^{2}}{1+D^{2}}\;\mathrm{and}\;R^{L}(\omega )=\frac{D(\tilde{E}-1)}{1+D^{2}}$$

where we recall that $\tilde{E}=E^{I}/E^{\infty }=\mu^{I}/\mu^{\infty }$ in the incompressible regime and *D* = *τω* is the Deborah number. Finally, with reference to §3, as it may be convenient to scale time or frequency on a characteristic time *τ*_*c*_ one can also define (analogously to (3.9))

5.33 $${\tilde{R}}^{S}(\tilde{D})=\frac{1+\tilde{E}{\left(\tilde{D}\tilde{\tau }\right)}^{2}}{1+{\left(\tilde{D}\tilde{\tau }\right)}^{2}}\;\mathrm{and}\;{\tilde{R}}^{L}(\tilde{D})=\frac{\left(\tilde{D}\tilde{\tau })(\tilde{E}-1\right)}{1+{\left(\tilde{D}\tilde{\tau }\right)}^{2}}$$

where $\tilde{D}=\omega \tau_{c}$, $\tilde{\tau }=\tau /\tau_{c}$ and given that this is merely a rescaling, these can directly replace $R^{S}$ and $R^{L}$ in (5.31). The storage and loss moduli associated with the perturbation are therefore

5.34 $$E_{*}^{S}(\lambda ,\tilde{D})=A(\lambda ){\tilde{R}}^{S}(\tilde{D})\;\mathrm{and}\;E_{*}^{L}(\lambda ,\tilde{D})=A(\lambda ){\tilde{R}}^{L}(\tilde{D}).$$

**Figure 4.**
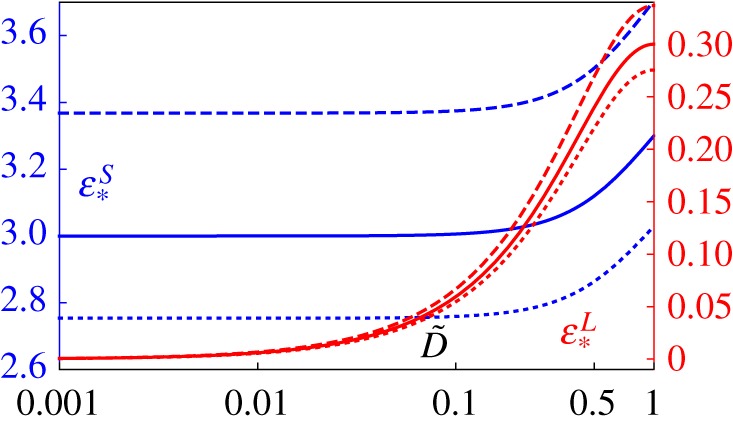
Illustrating the variation of the storage and loss moduli associated with longitudinal waves in a pre-stressed homogeneous thin rod as a function of non-dimensional frequency $\tilde{D}$. Different pre-stress levels are plotted for a Mooney–Rivlin material with *γ* = 0 and for stretches λ = 1 (solid), λ = 0.9 (dashed) and λ = 1.1 (dotted) for parameter values of the modulus contrast $\tilde{E}=6/5$ and $\tilde{\tau }=1$. Frequency-deformation separation for homogeneous QLV materials means that curves are simply scaled at different stretch levels. (Online version in colour.)

Compare (5.31) with the incremental case without viscoelasticity, (5.9). Notably, in this quasi-static regime one can therefore conclude that *homogeneous* QLV materials lead to small amplitude perturbations that exhibit *frequency-deformation separability* [53,54]. The deformation dependence is purely associated with the incremental modulus A(λ) which can be determined from the hyperelastic theory and the frequency dependence can be determined from the unstressed scenario. This is a consequence of the assumptions on $t\gg t_{1}\gg \bar{t}$ and the QLV model. In figure 4, we illustrate this by showing that pre-stress merely scales the storage and loss moduli associated with longitudinal vibrations of a QLV material that is under uniaxial tension.

## 6. Effective low-frequency viscoelastic waves in a pre-stressed inhomogeneous rod

The equations governing the incremental response of a thin *homogeneous* rod have been derived in both the hyperelastic and QLV scenarios. We now illustrate the effective low-frequency response of an *inhomogeneous* incompressible thin rod of the same geometry as that described in §3 but now under the quasi-static finite uniaxial deformation as described in §4c. We first consider the response in the absence of loss before moving on to the problem of main interest in this paper. Given that the material is incompressible *ρ*^0^ = *ρ* throughout and furthermore the volume fraction of each phase remains fixed during deformation, i.e. *ϕ*^0^ = *ϕ*.

### (a) Elastic waves

The equation governing longitudinal elastic waves in each phase is (5.9) with phase modulus $A_{r}$ and density *ρ*_*r*_. Non-dimensionalizing as per §3, the governing equation for incremental longitudinal waves is then given by

6.1 $$\frac{\partial \;}{\partial \tilde{x}}\left(\tilde{A}({\tilde{x}}_{1})\frac{\partial {\tilde{u}}_{1}}{\partial {\tilde{x}}_{1}}\right)+\epsilon^{2}\tilde{\rho }({\tilde{x}}_{1}){\tilde{u}}_{1}=0,$$

where $\tilde{A}=A/E_{c},\tilde{\rho }=\rho /\rho_{c}$ are piecewise constant.

Since the form of (6.1) is entirely equivalent to that in (3.6), the homogenization process can proceed in precisely the same manner so that we can determine the effective Young's modulus, stressing that this holds in the *elastic* regime but now in a pre-stressed state. This allows us to determine the influence of pre-stress on the effective wavespeed of the superposed wave. We can employ formula (3.10) for the effective Young's modulus with the only modifications being that we now use the incremental modulus in place of E, noting that (for now at least) the incremental Young's modulus is purely elastic. Therefore, the effective incremental Young's modulus (scaled on *E*_*c*_) for a two-phase periodic composite rod is straightforwardly defined as

6.2 $${\tilde{A}}_{*}=\frac{{\tilde{A}}_{2}{\tilde{A}}_{1}}{\phi {\tilde{A}}_{2}+(1-\phi ){\tilde{A}}_{1}}.$$

The effective mass density in the stressed state is simply the arithmetic mean:

6.3 $${\tilde{\rho }}_{*}=\phi {\tilde{\rho }}_{1}+(1-\phi ){\tilde{\rho }}_{2}$$

which we note is scaled on *ρ*_*c*_.

We consider examples where phases are Mooney–Rivlin as already defined in (4.22) as well as the Yeoh model, i.e.

6.4 $$W_{Y}=\frac{\mu^{\infty }}{2}\left(I_{1}-3\right)+\alpha \frac{\mu^{\infty }}{4}{\left(I_{1}-3\right)}^{2},$$

where *α* > 0. In this Yeoh strain energy case, the incremental modulus A as defined in (5.8) is

6.5 $$\begin{array}{r} A=\frac{\mu^{\infty }}{\lambda^{2}}\left[6\alpha +\lambda \left(\lambda^{3}+2+3\alpha \left(\lambda^{5}-\lambda^{3}-2\right)\right)\right]. \end{array}$$

Upon denoting ${\tilde{\mu }}_{r}=\mu_{r}/E_{c}$, *r* = 1, 2, we set the dimensionless elastic constants

6.6 $${\tilde{\mu }}_{1}^{\infty }=5\;\mathrm{and}\;{\tilde{\mu }}_{2}^{\infty }=3$$

and we also note again that in the incompressible scenario *E*^∞^ = 3*μ*^∞^.

**Figure 5.**
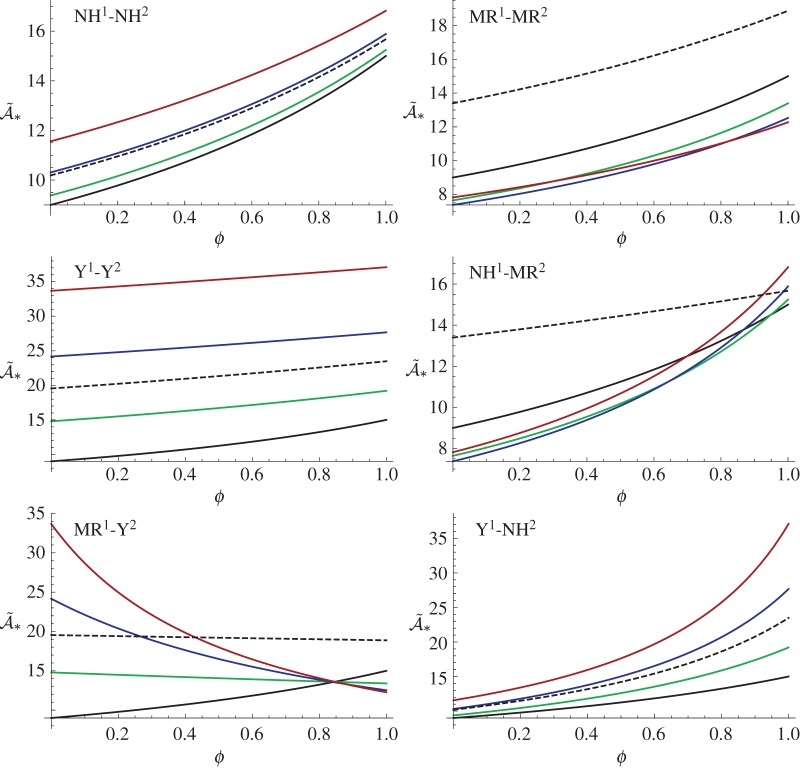
Predictions of ${\tilde{A}}_{*}$ versus *ϕ* when phase 1 and phase 2 are described by SEFs *W*_NH_ (*γ* = 1/2), *W*_MR_ (*γ* = 0) and Yeoh (*α* = 2) as denoted by NH, MR and Y, with a subscript 1 or 2, respectively, for an imposed stress $\tilde{T}=0$ (black solid line), $\tilde{T}=2$ (green solid line), $\tilde{T}=4$ (blue solid line), $\tilde{T}=6$ (red solid line), $\tilde{T}=-3$ (black dashed line). (Online version in colour.)

In figure 5, we plot ${\tilde{A}}_{*}$ as a function of *ϕ*, the volume fraction of phase 1, for a range of strain energy function combinations. We note in particular the sensitivity of the behaviour to the choice of strain energy. For the parameter regime illustrated, it is noteworthy that for all cases except one, pre-stress tends to stiffen the effective material response as additional phase 1 material is added to the inhomogeneous medium. The anomaly is the combination of Mooney–Rivlin (phase 1) and Yeoh (phase 2) for which pre-stress appears to have a softening effect for increasing *ϕ*. This indicates that the Yeoh model becomes relatively stiffer (compared to Mooney–Rivlin) for a given $\tilde{T}$ and therefore additional Mooney–Rivlin material only serves to soften the response. This macroscopic effect arises due to the combination of incremental moduli (5.10) and (6.5) in the harmonic mean form (6.2).

**Figure 6.**
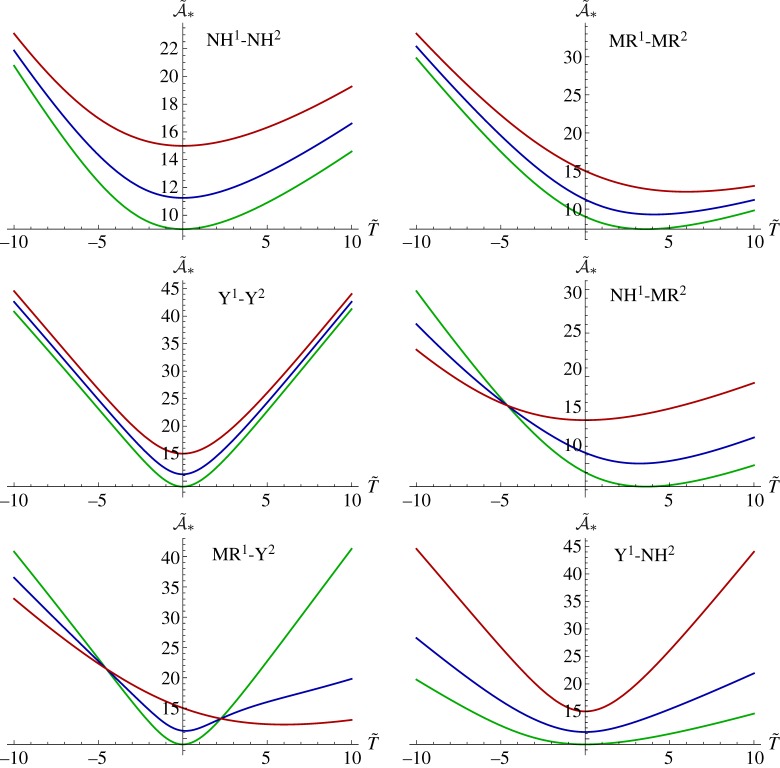
Predictions of ${\tilde{A}}_{*}$ versus $\tilde{T}$ when phase 1 and phase 2 are described by SEFs *W*_NH_ (*γ* = 1/2), *W*_MR_ (*γ* = 0) and Yeoh (*α* = 2) as denoted by NH, MR and Y, with a subscript 1 or 2, respectively, for an imposed *ϕ* = 0 (green line), *ϕ* = 0.5 (blue line), *ϕ* = 1 (red line). (Online version in colour.)

In figure 6, for the same range of strain energy combinations as in figure 5, we illustrate the specific response to tension for the case when *ϕ* = 0, 0.5 and 1 illustrating the ability to tune the incremental Young's modulus extremely effectively by combining nonlinear materials with pre-deformation.

We limit our discussion here given that this effect is well known and move on to the case of dominant interest—the influence of viscoelasticity.

### (b) Viscoelastic waves

Proceeding now to the *viscoelastic* scenario and for the regime of interest here, we employ (5.31) as the equation governing longitudinal wave propagation in each phase of the inhomogeneous rod.

Employing the notation used above, we can therefore define the pre-stressed incremental (frequency dependent) viscoelastic modulus in the *r*th phase as in (5.34), i.e. $E_{r}=A_{r}{\tilde{R}}_{r}$ (noting now the absence of the superscript ‘0’ as was employed for the unstressed modulus) and thus write (5.31) in an appropriate form for the inhomogeneous rod

6.7 $$\frac{\partial \;}{\partial x_{1}}\left(E(x_{1})\frac{\partial u_{1}}{\partial x_{1}}\right)+\omega^{2}\rho (x_{1})u_{1}=0$$

noting that now E depends on *x*_1_ and also on pre-stress. This equation is then non-dimensionalized by choosing an appropriate *E*_*c*_ and *ρ*_*c*_, to yield

6.8 $$\frac{\partial \;}{\partial {\tilde{x}}_{1}}\left(\tilde{E}({\tilde{x}}_{1})\frac{\partial {\tilde{u}}_{1}}{\partial {\tilde{x}}_{1}}\right)+\epsilon^{2}\tilde{\rho }({\tilde{x}}_{1}){\tilde{u}}_{1}=0,$$

where *ϵ* = *k*_*c*_*a* is defined as above. We can now determine the effective properties given that we have the equation in our canonical form. This allows us to write down the effective Young's modulus in the transform domain in the form

6.9 $${\tilde{E}}^{*}=\frac{{\tilde{E}}_{1}{\tilde{E}}_{2}}{(1-\phi ){\tilde{E}}_{1}+\phi {\tilde{E}}_{2}}$$

and we note that deformation and relaxation effects are clearly *not* separable in (6.9). This simple case serves to illustrate that even for the simplest of microstructural geometries one finds that

**Figure 7.**
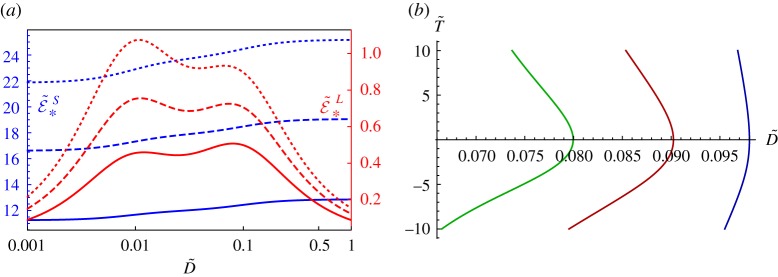
Illustrating the case when both phases behave quasi-statically as nonlinear elastic neo-Hookean materials. In (*a*), the effective incremental loss and storage moduli are plotted at $\tilde{T}=0$ (solid), $\tilde{T}=10$ (dashed) and $\tilde{T}=-10$ (dotted) and parameters employed are as in (3.12). In (*b*), we plot the value of $\tilde{D}$ for which loss modulus has a local maximum as a function of $\tilde{T}$, when ${\tilde{\mu }}_{1}^{\infty }=5,{\tilde{\mu }}_{2}^{\infty }=3$ (green line), ${\tilde{\mu }}_{1}^{\infty }=10,{\tilde{\mu }}_{2}^{\infty }=3$ (red line) and ${\tilde{\mu }}_{1}^{\infty }=50,{\tilde{\mu }}_{2}^{\infty }=3$ (blue line). (Online version in colour.)

6.10 $${\tilde{E}}^{*}\neq C(\lambda )R(\omega )$$

for some deformation dependent function *C*(λ). This form has been employed as a phenomenological expression for the form of effective strain-dependent relaxation of inhomogeneous materials by e.g. [53,54]. Expression (6.9) is exact in the asymptotic regime of interest here for low-frequency propagation in thin rods. In figure 7*a*, we illustrate the stretch dependence in the case of an inhomogeneous rod whose phases behave quasi-statically as neo-Hookean materials with long-term moduli defined in (3.12), noting that *μ*^∞^ = *E*^∞^/3 in the definition of the strain energy functions. Viscoelastic properties of the phases are as defined in (3.12). We plot the effective incremental loss and storage moduli associated with the unstressed and stressed ($\tilde{T}=\pm 10$) curves. It should be noted that the pre-stress stiffens the effect across a broad range of frequencies (as should be expected) but more importantly, this effect is non-uniform across the frequency spectrum, leading to the conclusion that frequency-deformation separability does not hold for the inhomogeneous material considered here. In figure 7*b*, we plot the value of the Deborah number $\tilde{D}$ at which the loss modulus achieves its local maximum close to $\tilde{D}=0.1$ with varying $\tilde{T}$ and for increasing phase 1 shear modulus: ${\tilde{\mu }}_{1}^{\infty }=5$ (green), ${\tilde{\mu }}_{1}^{\infty }=10$ (red) and ${\tilde{\mu }}_{1}^{\infty }=50$ (blue) when ${\tilde{\mu }}_{2}^{\infty }=3$. These results are in stark contrast to the homogeneous bar case as depicted in figure 4 where the spatial homogeneity ensures that the frequency-deformation separation remains.

**Figure 8.**
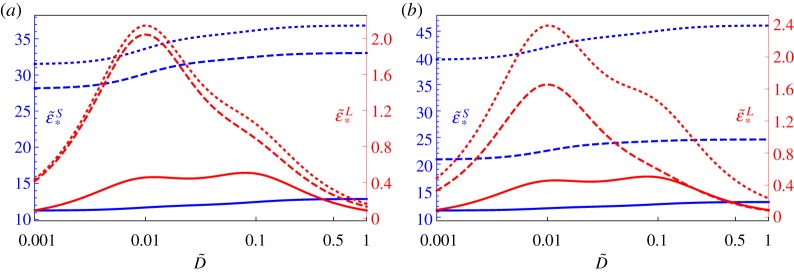
Illustrating the effective incremental loss and storage moduli in the case when both phases behave quasi-statically as nonlinear elastic materials with phase 2 as a Yeoh material. In (*a*), phase 1 is neo-Hookean and in (*b*) phase 1 is Mooney–Rivlin with *γ* = 0. Curves correspond to $\tilde{T}=0$ (solid), $\tilde{T}=10$ (dashed) and $\tilde{T}=-10$ (dotted) and parameters employed are as in (3.12). (Online version in colour.)

To further illustrate the lack of frequency-deformation separability, the effective incremental loss and storage moduli are plotted in figure 8 for the cases when the quasi-static nonlinear elastic behaviour of phase 2 is of Yeoh type and phase 1 is (*a*) neo-Hookean and (*b*) Mooney–Rivlin with *γ* = 0, together with the parameter set (3.12). The striking effect of the lack of frequency-deformation separability is particularly evident: this effect is emphasized here when the phases possess different strain energy functions.

## 7. Conclusion

A methodology and formulation has been presented in order to determine the effective incremental dynamic (loss and storage) moduli associated with pre-stressed inhomogeneous nonlinear materials. In particular, it is noted that frequently relaxation times of such soft materials are long and therefore these media are very often frequency dependent even at ‘low’ frequencies where classical homogenization methods would normally give properties that are independent of frequency. It is shown that the effective incremental moduli are very sensitive to the choice of strain energy functions of the constituent phases. Furthermore, and perhaps most importantly, it is shown that even when constituent phases are assumed to be time-deformation separable in their individual constitutive response, the resulting effective behaviour of the thin inhomogeneous rod has strong frequency-deformation (and hence time-deformation) coupling. We note that at higher frequencies, such periodic materials would act as soft-phononic crystals. One could extend the above analysis to that context but the fully dispersive rod theory would then have to be developed, taking into account lateral inertia and other effects that become important at higher frequencies.

## Data accessibility

All results can be obtained from information provided in the manuscript.

## Appendix A. Transform theory

Given the form

A 1 $$\hat{\sigma }(t)=\int_{-\infty }^{t}\hat{G}(t-\tau ):\frac{\partial e}{\partial \tau }(\tau )\;d\tau$$

apply the Fourier transform, defined on some tensor function $\hat{f}(t)$ by

A 2 $$f(\omega )=\int_{-\infty }^{\infty }\hat{f}(t)\;e^{i\omega t}\;dt$$

in order to find that, upon interchanging orders of integration,

A 3 $$\sigma (\omega )=\int_{-\infty }^{\infty }\int_{\tau }^{\infty }\hat{G}(t-\tau ):\frac{\partial e}{\partial \tau }(\tau )\;e^{i\omega t}\;dt\;d\tau$$

and finally upon making the change of variable *u* = *t* − *τ*, one arrives at

A 4 $$\sigma (\omega )=-i\omega G^{+}(\omega )e(\omega )$$

noting standard properties of transforms of derivatives and with the definition

A 5 $$f^{+}(\omega )=\int_{0}^{\infty }\hat{f}(t)\;dt.$$
